# Renal Carcinoma Is Associated With Increased Risk of Coronavirus Infections

**DOI:** 10.3389/fmolb.2020.579422

**Published:** 2020-11-20

**Authors:** Satyendra C. Tripathi, Vishwajit Deshmukh, Chad J. Creighton, Ashlesh Patil

**Affiliations:** ^1^Department of Biochemistry, All India Institute of Medical Sciences, Nagpur, India; ^2^Bioinformatics Data Analysis Unit (BDAU), All India Institute of Medical Sciences, Nagpur, India; ^3^Department of Anatomy, All India Institute of Medical Sciences, Nagpur, India; ^4^Department of Medicine and Dan L Duncan Comprehensive Cancer Centre, Baylor College of Medicine, Houston, TX, United States; ^5^Department of Physiology, All India Institute of Medical Sciences, Nagpur, India

**Keywords:** coronavirus, cancer, kidney, ACE2, DPP4, tmprss2

## Abstract

**Background:** The current COVID-19 pandemic has affected most severely people with old age, or with comorbidities like hypertension, diabetes mellitus, and cancer. Cancer patients are twice more likely to contract the disease because of the malignancy or treatment-related immunosuppression; hence identification of the vulnerable population among these patients is essential.

**Method:** We took a bioinformatics approach to analyze the gene and protein expression data of these coronavirus receptors (DPP4, ANPEP, ENPEP, TMPRSS2) in human normal and cancer tissues of multiple organs including the brain, liver, kidney, heart, lung, skin, GI tract, pancreas, endocrine tissues, and the reproductive organs. RNA-Seq data from The Cancer Genome Atlas (TCGA) and GTeX databases were used for extensive profiling analysis of these receptors across 9,736 tumors and 8,587 normal tissues comparing coronavirus receptors. Protein expression from immunohistochemistry data was assessed from The Human Protein Atlas database including 144 samples, corresponding to 48 different normal human tissue types, and 432 tumor samples from 216 different cancer patients. The correlations between immune cell infiltration, chemokine, and cytokines were investigated via Tumor Immune Estimation Resource (TIMER) and TCGA.

**Result:** We found that among all, renal tumor and normal tissues exhibited increased levels of ACE2, DPP4, ANPEP, and ENPEP. Our results revealed that TMPRSS2 may not be the co-receptor for coronavirus infection in renal carcinoma patients. The other receptors DPP4, ANPEP, and ENPEP may act as the compensatory receptor proteins to help ACE2. The receptors' expression levels were variable in different tumor stage, molecular, and immune subtypes of renal carcinoma. Intriguingly, in clear cell renal cell carcinomas, coronavirus receptors were associated with high immune infiltration, markers of immunosuppression, and T cell exhaustion.

**Conclusion:** Our study indicates that CoV receptors may play an important role in modulating the immune infiltrate and hence cellular immunity in renal carcinoma. As our current knowledge of pathogenic mechanisms will improve, it may help us in designing focused therapeutic approaches.

## Introduction

Coronavirus (CoV) disease-2019 (COVID-19) has been declared as a pandemic by the World Health Organization (WHO) after the outbreak of severe acute respiratory syndrome-CoV-2 (SARS-CoV-2) (Ng et al., 2020). The primary host for the SARS-CoV-2 has been identified as bats and the terminal host as humans (Khan et al., 2020). The related research revealed that SARS-CoV und SARS-CoV-2 share ~76% of amino acid identity (Wang et al., 2020). The primary symptoms include fever, dry cough, dyspnea, diarrhea, myalgia, headache, hyposmia, and with common complications like acute respiratory distress (29%), acute cardiac injury (12%), and acute renal injury (7%) (Huang et al., 2020).

It has been well-established that CoVs require the ACE2 or DPP4 receptors for entry into the host cells (Seys et al., 2018; Walls et al., 2020; Wrapp et al., 2020). The organs or the cells expressing ACE2 are more vulnerable to the CoV infection (Glowacka et al., 2011). The viral entry in the host cell depends on the SARS-CoV receptor ACE2 for binding and requires the TMPRSS2 for priming and also relies on TMPRSS2 activity (Hoffman et al., 2020; Patil et al., 2020). Therefore, it is speculated that other auxiliary proteins or co-receptors might facilitate the entry of CoVs in the host cells. These co-receptors or auxiliary proteins include TMPRSS2, ANPEP, ENPEP (Qi et al., 2020). Therefore, it has been proposed that organs representing the co-expression of co-receptors or auxiliary proteins such as TMPRSS2, ANPEP, or ENPEP for ACE2 and DPP4 are more susceptible for the viral entry replication and severity of the disease. The thought of extrapulmonary spread is not evitable due to the presence of these receptors and co-receptors.

The prominent population with increased risk of virus infection are older patients and those associated with comorbidities such as hypertension, diabetes mellitus, chronic kidney disease, COPD, and cancers (Sun et al., 2020). Viral infection with comorbidities is responsible for higher mortality. According to Lee et al. cancer patients are twice more likely to contract the infection than the normal population (Lee et al., 2020). Patients who received chemotherapy or surgery within the 30 days before the COVID-19 pandemic have more risk of infection than the patients who had not undergone chemotherapy or surgery (Sharma et al., 2020). According to an analysis of Italian patients published in March, 20% of those who died from COVID-19 in the country had active cancer (Liang et al., 2020). Notably, the guidelines for cancer patients during the COVID-19 pandemic focus on lung cancer patients undergoing active chemotherapy or radical radiotherapy, and on patients with blood cancers (Burki, 2020). The association of these receptors with the pathogenicity of the solid tumors is still to be solved.

In the present study, we investigated molecular profiling data of the various proteins required for the entry of the CoVs in normal tissues and cancer tissues. Immunological aspects of the study of pathogenesis cannot be overlooked. Therefore, we also explored an immune perspective concerning cancer. Understanding the usage of the multiplicity of receptors and co-receptors by the various CoVs can open new avenues for understanding the pathogenesis and development of intervention strategies for cancer patients.

## Results

### Expression Profiles of CoV Receptors in Normal and Tumor Tissues

**Figure 1:**
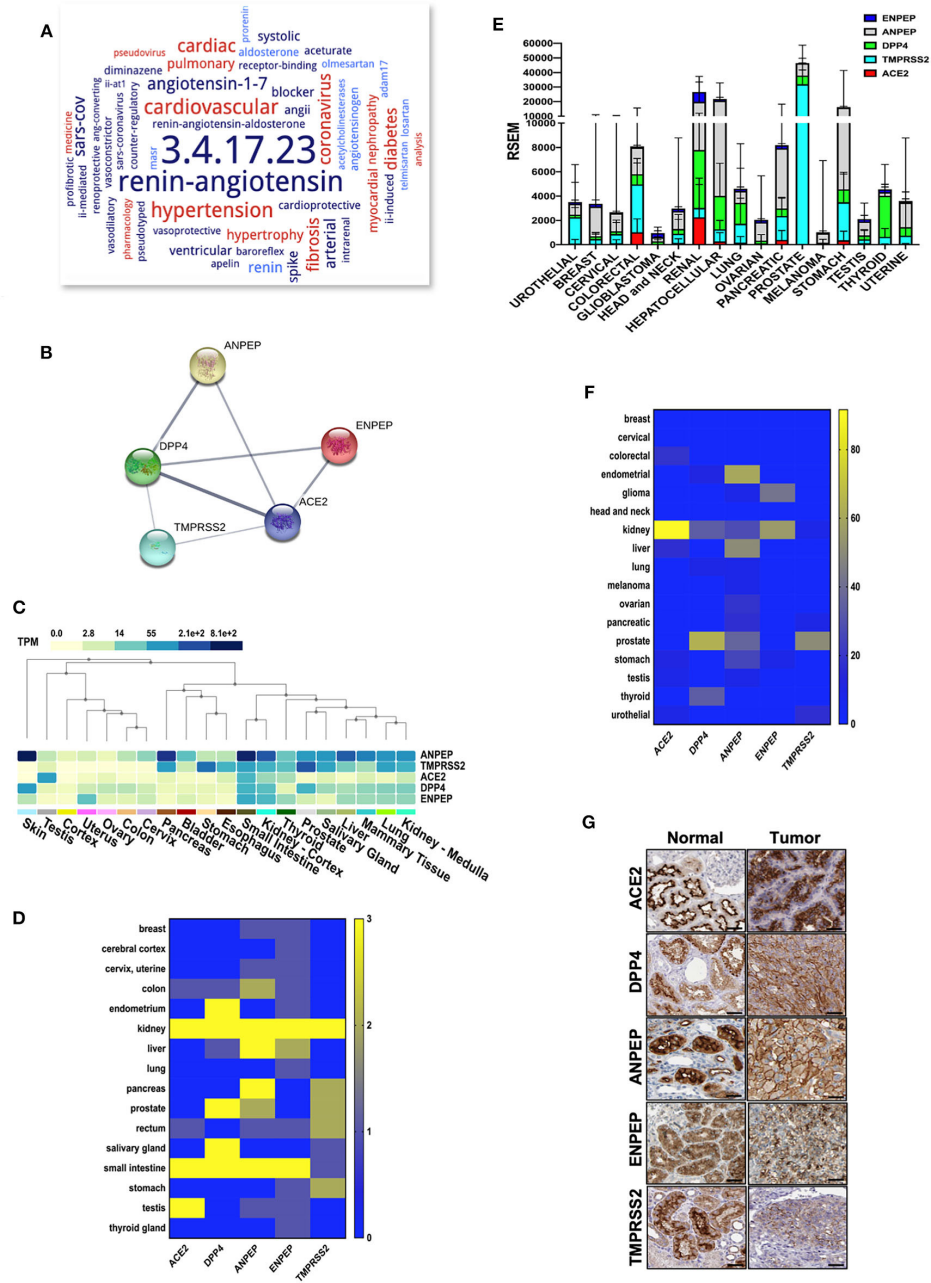
Expression of coronavirus receptors in human tissues. **(A)** The word cloud of various attributes associated with ACE2. Analysis identified the ACE2 enzyme functions and related disorders with the most prominent one appearing larger than the remaining words **(B)** All coronavirus receptors (ACE2, DPP4, TMPRSS2, ANPEP, and ENPEP) were used with high confidence (0.8) evidence from experimental protein–protein interaction (blue lines) databases for STRING analysis. Each Node labeled with the encoding gene symbol represents a protein. **(C)** Heatmap of mRNA expression extracted from the GTEx database for coronavirus receptors in various human normal tissues and organs (*n* = 8,587). Kidney and small intestine tissues showed enriched mRNA expression of coronavirus receptors **(D)** Protein expression heatmap for coronavirus receptors in normal tissues (*n* = 144). Kidney and small intestine tissues exhibited increased protein expression in concordance with mRNA. **(E)** The mRNA expression profile of coronavirus receptors extracted from TCGA database in different cancer tissues (*n* = 9,736) Renal tumors exhibited the highest expression of all coronavirus receptors except TMPRSS2. **(F)** Protein expression heatmap for coronavirus receptors in cancer tissues (*n* = 432). Renal carcinoma tissues exhibited increased protein expression of ACE2 and ENPEP in concordance with mRNA data. **(G)** Representative images of immunohistochemistry images of coronavirus receptors in normal and renal carcinoma tissues (source: The Human Protein Atlas; https://www.proteinatlas.org/humanproteome). TMPRSS2 expression is reduced as compared to other receptors in cancer tissues. Scale bar = 50 um. ACE2, angiotensin-converting enzyme II; DPP4, Dipeptidyl-peptidase 4; ANPEP, Alanyl Aminopeptidase; ENPEP, Glutamyl Aminopeptidase; TMPRSS2, Transmembrane Serine Protease 2.

SARS-Cov-2 requires ACE2 as its receptor for host cell entry, an enzyme that is involved in various biological functions primarily in the renin-angiotensin system (Figure 1A). DPP4, ANPEP, ENPEP, and TMPRSS2 have also been proposed as co-receptors to initiate SARS-CoV-2 and other coronavirus infections (Qi et al., 2020). STRING pathway analysis revealed a high confident interaction between these proteins, which have peptidase activity and are involved in the angiotensin system, peptide metabolism, and viral entry into the host cell (Figure 1B and Table 1). Hence, we extracted the RNA and protein expression data available on public databases for all these receptors in 8,587 healthy tissues and 9,736 tumors. We found enrichment of ACE2 at RNA levels in testis, small intestine, and kidney (Figure 1C). Along with ACE2, enrichment of RNAs for the other four co-receptors occurred in normal lung, mammary, liver, prostate, thyroid, head and neck tissues, small intestine, and kidney tissues (Figure 1C). The protein expression levels of ACE2 and co-receptors showed a similar expression pattern as to their RNA (Figure 1D). Renal tumors exhibited the highest expression of ACE2 receptor followed by gastrointestinal cancers such as colorectal, pancreatic, and stomach cancer (Figure 1E and Supplementary Figure 1). DPP4, ANPEP, and ENPEP RNA expression were also elevated in renal tumors compared to other cancer tissues (Figure 1E and Supplementary Figure 1). Of note, TMPRSS2 RNA expression was highest in prostate cancer tissues, whereas renal tumors were featured among the lowest expressing tissues. TCGA data analysis also revealed increased RNA expression of all receptors except TMPRSS2 in renal tumor tissues as compared to their adjacent normal (Supplementary Figure 2). We found that protein level data was also in concordance to RNA expression data in tumors (Figure 1F). Renal cancer expressed higher percent positivity of all receptors except TMPRSS2, which exhibited the highest percent positivity in prostate tumor tissues. Immunohistochemical analysis of these coronavirus receptors revealed that TMPRSS2 levels decrease during renal carcinoma as compared to normal renal tissues (Figure 1G).

**Table 1:** Functional and molecular process related to coronavirus receptors.

**GO term**	**Functional process**	**FDR**	**Proteins**
GO:0008238	Exopeptidase activity	1.79E-07	ACE2, ANPEP, DPP4, ENPEP
GO:0070011	Peptidase activity	5.41E-07	ACE2, ANPEP, DPP4, ENPEP, TMPRSS2
GO:0004177	Aminopeptidase activity	1.23E-06	ANPEP, DPP4, ENPEP
GO:0008235	Metalloexopeptidase activity	2.07E-06	ACE2, ANPEP, ENPEP
GO:0001618	Virus receptor activity	3.83E-06	ACE2, ANPEP, DPP4
GO:0070006	Metalloaminopeptidase activity	5.08E-05	ANPEP, ENPEP
GO:0004175	endopeptidase activity	0.00026	ACE2, DPP4, TMPRSS2
GO:0008270	Zinc ion binding	0.0019	ACE2, ANPEP, ENPEP
GO:0004252	Serine-Type endopeptidase activity	0.0023	DPP4, TMPRSS2
GO:0042277	Peptide binding	0.004	ANPEP, ENPEP
**GO Term**	**Molecular process**	**FDR**	**Proteins**
GO:0002003	Angiotensin maturation	0.00025	ACE2, ENPEP
GO:0006508	Proteolysis	0.00025	ACE2, ANPEP, DPP4, ENPEP, TMPRSS2
GO:0008217	Regulation of blood pressure	0.00025	ACE2, ANPEP, ENPEP
GO:0016485	Protein processing	0.00025	ACE2, ENPEP, TMPRSS2
GO:0046718	Viral entry into host cell	0.00025	ACE2, ANPEP, DPP4
GO:0043171	Peptide catabolic process	0.00047	ANPEP, ENPEP
GO:0006518	Peptide metabolic process	0.0019	ACE2, ANPEP, ENPEP
GO:0010817	Regulation of hormone levels	0.002	ACE2, DPP4, ENPEP
GO:0001525	Angiogenesis	0.0183	ANPEP, ENPEP

### Analysis of CoV Receptors in Renal Carcinoma

**Figure 2:**
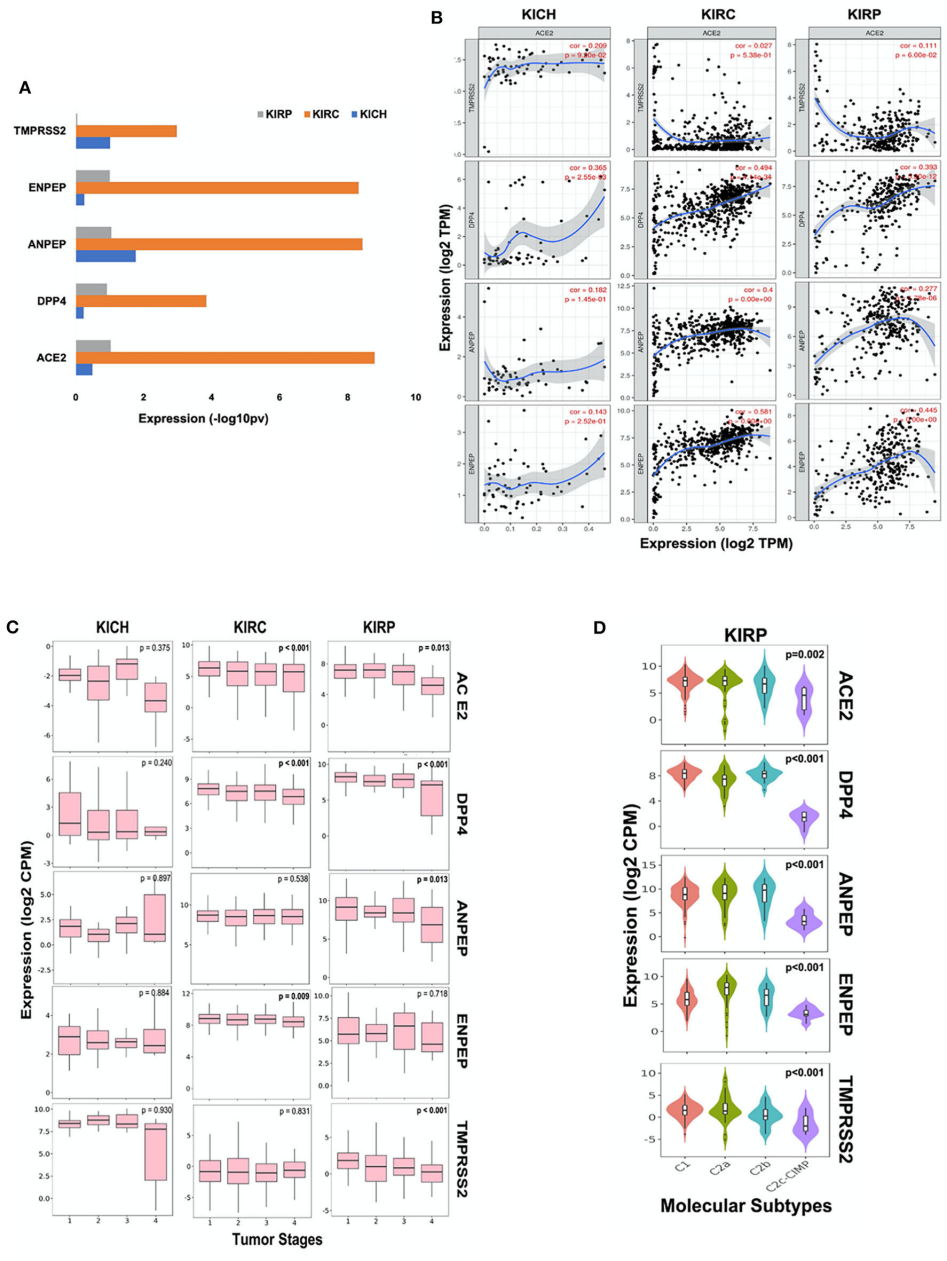
Coronavirus receptors in Renal carcinoma. **(A)** The bar graph depicting a comparison of coronavirus receptor expression in different subtypes (KIRC, *n* = 515; KIRP, *n* = 279; KICH, *n* = 65) of renal carcinoma. KIRC exhibited the highest expression of all coronavirus receptors as compared to whereas KIRP exhibited negligible expression of TMPSS2. **(B)** Correlation scatterplot of ACE2 with DPP4, ANPEP, ENPEP, and TMPRSS2 in all renal carcinoma subtypes. ACE2 was significantly correlated with DPP4 in all renal carcinoma subtypes. **(C)** Boxplot for coronavirus receptors expression in different tumor stages. ACE2 and DPP4 exhibited significant negative correlation with tumor stages in KIRC (*n* = 515) and KIRP (*n* = 279). **(D)** Violin plot for coronavirus receptors expression across various molecular subtypes in KIRP tumors (*n* = 279). All coronavirus receptors showed significantly increased expression in C1, C2a, and C2b subtypes compared to the C2c-CIMP subtype. KIRP, Kidney renal papillary cell carcinoma; KIRC, Kidney renal clear cell carcinoma; KICH, Kidney renal chromophobe.

We further analyzed the expression of ACE2, ANPEP, ENPEP, DPP4, and TMPRSS2 gene in renal carcinoma tissues (Supplementary Figure 3A). We observed increased expression of these five coronavirus receptor genes in renal clear cell carcinoma (KIRC) as compared to renal papillary carcinoma (KIRP) and renal chromophobe (KICH). ACE2, DPP4, and ENPEP exhibited the lowest expression in KICH, whereas TMPRSS2 and ANPEP were decreased in KIRP subtypes (Figure 2A). We further analyzed the correlation of ACE2 with all four receptors in each renal cancer types. We observed a statistically significant correlation of ACE2 with DPP4 in KICH (*p* < 0.01, ρ = 0.365), KIRC (*p* < 0.001, ρ = 0.494), KIRP (*p* < 0.001, ρ = 0.393), and also with ANPEP in KIRP (*p* < 0.001, ρ = 0.277; Figure 2B).

Analysis of different tumor stages of renal carcinoma subtypes revealed a statistically significant (*p* < 0.01) negative Spearman's correlation of ACE2 and DPP4 with increasing stages of KIRC and KIRP tumors. However, TMPRSS2 exhibited very low expression levels, and also negatively correlated with tumor stages in KIRP tumor tissues along with ANPEP (ρ = −0.153, *p* < 0.05). ENPEP only showed a significant (ρ = −0.112, *p* < 0.01) negative correlation with tumor stages in KIRC tumors (Figure 2C). As molecular subtypes of KIRP are well-defined, we also analyzed the expression pattern of these receptors in various molecular subtypes. We observed that all CoV receptors showed significantly (*p* < 0.001, Kruskal–Wallis test) increased expression in C1, C2a, and C2b subtypes compared to the C2c-CIMP subtype of KIRP (subtype with high DNA methylation) (Figure 2D).

### Association of CoV Receptors With Immune Cell Signature in Renal Carcinoma Immune Subtypes

**Figure 3:**
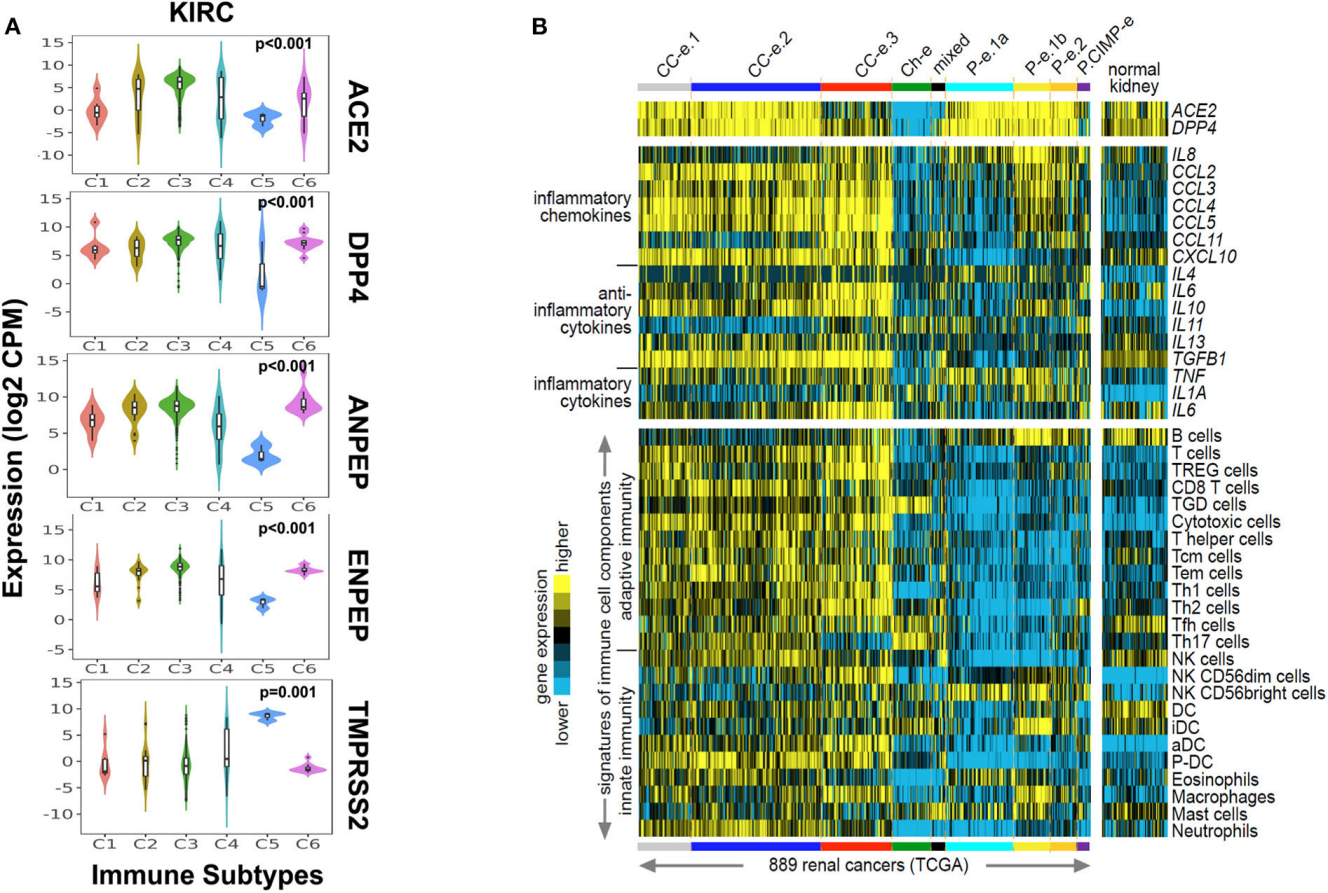
Immune signatures in renal carcinoma with coronavirus receptors expression. **(A)** Violin plot for coronavirus receptors expression across various immune subtypes in KIRC tumors (*n* = 515). TMPRSS2 exhibited the lowest expression as compared to other coronavirus receptors in C1–C4 and C6 immune subtypes. **(B)** Heatmaps of differential expression across renal cell carcinoma cases from TCGA dataset (Chen et al., 2016) for genes encoding ACE2 and DPP4 (top), genes encoding selected chemokines and cytokines (middle), and gene expression-based signatures of immune cell infiltrates (bottom). Cases are ordered by molecular subtype as defined previously (Chen et al., 2016). Three of these subtypes—CC–e.1, CC–e.2, and CC–e.3—are enriched for KIRC cases; four other subtypes—P–e.1a, P–e.1b, P–e.2, and P.CIMP-e—are enriched for KIRP cases; one subtype, Ch-e, is enriched for KICH cases; and one subtype (“mixed”) is not enriched for any of the above. TREG cells, regulatory T cells; TGD cells, T gamma delta cells; Tcm cells, T central memory cells; Tem cells, T effector memory cells; Tfh cells, T follicular helper cells; NK cells, natural killer cells; DC, dendritic cells; iDC, immature DCs; aDC, activated DCs; P-DC, plasmacytoid DCs; KIRP, Kidney renal papillary cell carcinoma; KIRC, Kidney renal clear cell carcinoma; KICH, Kidney renal chromophobe.

As CoV infection leads to immunological consequences, we investigated CoV receptors' expression across the immune subtypes of renal carcinoma. The immune subtypes—C1 (wound healing), C2 (IFN-gamma dominant), C3 (inflammatory), C4 (lymphocyte depleted), C5 (immunologically quiet), and C6 (TGF-b dominant)—are each present across sizable subsets of KIRP and KIRC tumors (Ru et al., 2019). We observed that, except for TMPRSS2, the other four CoV receptors were highly significantly (*p* < 0.001, Kruskal–Wallis test) expressed in C1–C4 and C6 immune subtypes of KIRC and KIRP tumors. Whereas, immune subtype C5 had a higher expression of TMPRSS2 (Figure 3A and Supplementary Figure 3B). We also analyzed the correlation between the CoV receptor genes and immune cell signatures, for each of the 32 cancer types in TCGA. The CoV receptors tended to show a high correlation with immune signatures in most cancer types, though ACE2 and TMPRSS2 exhibited weaker correlations than other receptors (Supplementary Figure 3C). Further, we specifically analyzed ACE2, which is a primary SARS-CoV receptor and DPP4, highly correlated to ACE2 across the kidney cancer subtypes along with immune cell signatures (innate and adaptive immunity, inflammatory cytokines, and chemokines) in 889 renal cancer samples and 409 normal kidney tissues. We observed that ACE2 and DPP4 exhibited increased expression in all molecular subtypes except KICH, CIMP subtype of KIRP, and CC-e.3 KIRC subtype (Figure 3B). We found that these receptors are highly correlated to the innate and adaptive immunity-related cells, as well as IL-10, IL-6, CXCL10, CCL2-CCL5, TGFB1 in KIRC tumors, whereas in KIPRP tumor tissues IL8, IL1A, and TNF were highly correlated (Figure 3B). Our results revealed that, in KIRC tumors, the expressions of various markers of exhausted T cells (CD137, PD1, CTLA4) and immunosuppressive microenvironment (PDL1, PDL2) were also significantly (*p* < 0.01, *t*-test) correlated to CoV receptors (Supplementary Figure 3D).

### Immune Infiltrate Status and Association With CoV Receptors in Renal Cancer

**Figure 4:**
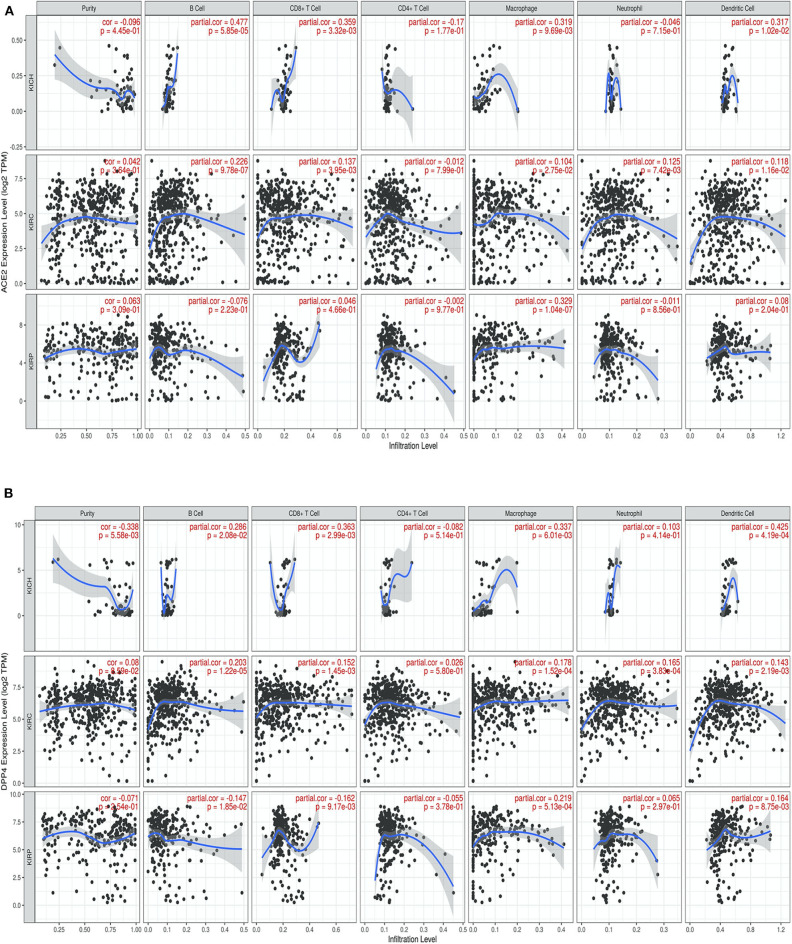
Correlation of coronavirus receptors and Tumor immune infiltrate **(A,B)** Correlation scatterplot of **(A)** ACE2 and **(B)** DPP4 with tumor purity and tumor immune infiltration of B cell, CD8+ T cell, CD4+ T cell, Macrophage, Neutrophil, and Dendritic cell in renal carcinoma (KIRC, *n* = 553; KIRP, *n* = 290; KICH, *n* = 66). ACE2 and DPP4 exhibited a positive correlation with infiltrating levels of B cells, CD8+ T cells, macrophages, neutrophils, and dendritic cells. KICH, Kidney renal chromophobe; KIRP, Kidney renal papillary cell carcinoma; KIRC, Kidney renal clear cell carcinoma.

We next investigated whether CoV receptor expression correlated with immune infiltration levels in renal carcinoma from TIMER. Notably, CoV receptors expression significantly correlated with infiltrating levels of various immune cells. In KIRC subtype, ACE2, DPP4, ANPEP, and ENPEP showed significant (*p* > 0.05), moderately positive Spearman's correlation with infiltrating levels of B cells, CD8+ T cells, macrophages, neutrophils, and dendritic cells (DCs) (Figure 4 and Supplementary Figure 3). ACE2 was only found to be significantly positively correlated to macrophage (*r* = 0.329, *p* = 1.04e-07) immune infiltrate in KIRP subtype (Figure 4A). On the other hand, DPP4 was significantly positively and negatively correlated to macrophage (*r* = 0.219, *p* = 5.13e-04) and B cell (*r* = −0.147, *p* = 1.85e-02), CD8+ T cell (*r* = 0.162, *p* = 9.17e-03) immune infiltrate in KIRP subtype (Figure 4B). We observed either negative or no correlation of TMPRSS2 expression with immune infiltrates in renal carcinoma subtypes (Supplementary Figure 4A). We also found a significant correlation of ANPEP and ENPEP with immune filtrate in KIRC as compared to KIRP tumors (Supplementary Figures 4B,C). Immune infiltrates of B cells, CD8+ T cells, Macrophages, and DCs significantly correlated with these receptors except TMPRSS2 in KICH tumors (Figure 4 and Supplementary Figure 4). These results strongly suggest a variable host immune response to CoV infection depending upon the renal carcinoma subtypes.

## Discussion

There is an increased risk of coronavirus related fatalities in the subpopulation with any underlying health conditions or comorbidities. The risk factors for an increased susceptibility for CoV infection include, but are not limited to, diabetes, heart disease, hypertension, chronic renal disease, chronic obstructive pulmonary disease, smoking, and cancer (Guan et al., 2020). Cancer patients especially those undergoing chemotherapy and other anti-cancer treatment have an increased risk of mortality due to CoV infection (Lee et al., 2020). The precise mechanism of increased severity in cancer remains unclear. In this study, we did landscape profiling of CoV receptors (viz ACE2, TMPRSS2, ANPEP, ENPEP, and DPP4) in various normal and cancer cells.

Our findings are in concordance with previous studies reporting ACE2 expression in stratified epithelial cells, colon, lung, liver, and kidney. Single-stranded RNA viruses can have multiple receptors for their host cell entry (Zhang et al., 2019). SARS-CoV utilizes ACE2, CD209, CLEC4G, and CLEC4M for its infection to host (Marzi et al., 2004; Yang et al., 2004; Gramberg et al., 2005; Wang et al., 2008). ACE2 has also been reported to be a co-receptor for SARS-CoV-2 where it is a key player with prolonged pathogenesis of COVID-19 (Zhou et al., 2020). A recent study has suggested a few other receptors such as DPP4, ANPEP, ENPEP, and TMPRSS2 as co-receptors/auxiliary proteins to complement ACE2 in initiating SAR-CoV-2 infection (Qi et al., 2020). DPP4, a known receptor for MERS-CoV may also act as an entry route for SARS-CoV-2 and can contribute toward the COVID-19 related adversities (Solerte et al., 2020; Vankadari and Wilce, 2020). Single nucleotide polymorphism of TMPRSS2 has also been proposed as a key factor in the pathogenesis of coronaviruses (Stopsack et al., 2020). ANPEP and ENPEP are not been reported yet to be associated with SARS-C0V-2. However, ENPEP is a key contributor in the renin-angiotensin system and ANPEP has been reported to be associated with previous coronavirus 229E (Yeager et al., 1992; Holmes et al., 2017). We analyzed the data available on the GTeX portal and observed the co-occurrence of these receptors in the small intestine and kidney at both RNA and protein levels.

Coronavirus infection is a multiorgan diseased condition and is not limited to the lungs. Extrapulmonary manifestations and spread is plausible considering the expression of coronavirus receptors in various organs. Few studies have reported low levels of ACE2, in lung parenchyma as compared to other normal tissues (Jia et al., 2005; Qi et al., 2020). Cell type specificity also exists for ACE2 expression, such as in lung expression is mainly in alveolar cells (Type 2 pneumocytes) and immune cells (B cells, T cells, or myeloid cells) (Qi et al., 2020). In kidneys, most of the cells of proximal collecting tubules and proximal straight tubules exhibit increased ACE2 expression. Coronavirus damages the tissue by direct virus-mediated cell damage or via dysregulation of the immune response and hyper inflammation leading to organ failure (Gupta et al., 2020). The extrapulmonary spread has been documented in SARS-Cov2 with histopathological studies showing organotropism of the coronavirus in renal, cardiac, neural, and gastrointestinal tissues (Puelles et al., 2020; Xiao et al., 2020).

Various evidence suggested that coronavirus can infect the human kidney directly. A postmortem analysis of six patients revealed the presence of severe acute tubular necrosis with the accumulation of SARS-CoV-2 nucleocapsid protein antigens in kidney tubules, which leads to kidney dysfunction (Diao et al., 2020). The differences between the multiorgan tropism of coronavirus could be explained by the variable affinity of these viral proteins toward their receptors. Hence, distributing the viral load in several organs, which may act as a viral reservoir (Perico et al., 2020). Another study using electron microscopy identified coronavirus particles in the proximal tubular epithelium and podocytes of autopsies of 26 patients (Su et al., 2020). Direct SARS-CoV-2 infection of the renal epithelium may also be potential pathways of kidney damage through mitochondrial dysfunction, tubular and endothelial injury, or protein leakage (Larsen et al., 2020; Ronco et al., 2020).

A substantial portion of COVID-19 patients also had GI symptoms with diarrhea as the first symptom suggesting that the GI tract might be a route of invasion and transmission of the virus (Gao et al., 2020; Song et al., 2020).

Cancer patients are always at higher risk of COVID-19 infection, and related case fatalities due to the compromised immune system increases comorbidities or treatment-related immunosuppression (Mehta et al., 2020; Tagliamento et al., 2020). We observed an increased expression of ACE2, DPP4, ANPEP, and ENPEP in renal tumor data available on TCGA followed by gastrointestinal cancers such as colorectal, pancreatic, and stomach cancer. This observation further raises the possibility that, along with lung cancer, patients with other types of cancer may also have elevated infection risk. Further analysis of renal carcinoma subtypes revealed that KIRP and KIRC tumors exhibit increased levels of DPP4, ANPEP, and ENPEP along with ACE2 receptors. We found that the expression of these receptors is inversely correlated to tumor stage and varies by molecular subtypes in renal carcinoma.

As host immune response is crucial to eradicating viral infection, immunological aspects related to these receptors cannot be overlooked. We observed that CoV receptors tend to show a high correlation with immune signatures in most cancer types. We further explored the possibility, whether CoV receptors are involved in modulating tumor immunity. Our analysis revealed for the first time that these receptors were correlated with immune cell infiltration in renal carcinoma. Our findings supported the immunoregulatory functions as follows: ACE2, DPP4, ANPEP, and ENPEP expression is closely related to the infiltration level of B cells, CD8+ T cell, macrophage, neutrophil, and dendritic cell. Cytokine storm has been well-defined and described with the pathogenesis of the disease. Chemokines are involved in several biological processes, such as the development of innate and acquired immunity, embryogenesis, and cancer metastasis (Coperchini et al., 2020). These chemokines along with cytokines recruit different immune cells (Poeta et al., 2019). We found that ACE2 and DPP4 were highly expressed and significantly correlated to the innate and adaptive immunity-related cells, as well as IL-10, IL-6, CXCL10, CCL2-CCL5, TGFB1 in KIRC tumors only. Upregulation of CXCL10 can enhance the levels of Tumor-infiltrating CD8+ T cell and natural killer cells (Humblin and Kamphorst, 2019; Kikuchi et al., 2019; Petty et al., 2019). T regulatory cells and macrophages get recruited by CCL5 (Wang et al., 2017; Walens et al., 2019). These results indicate that CoV receptors can play an important role in cellular immunity by modulating the immune infiltrate via cytokines and chemokines secretion.

Cancer cells also can evade the immune system by promoting T cell dysfunction and exhaustion (Jiang et al., 2015; Thommen and Schumacher, 2018). We analyzed the expression of inhibitory immune-checkpoint molecules in renal carcinoma subtypes to understand the dysregulation of the tumor microenvironment. Our results revealed that the expressions of various markers of exhausted T cells (CD137, PD1, CTLA4) and immunosuppressive microenvironment (PDL1, PDL2) are highly correlated to CoV receptors in KIRC tumors. Therefore, targeting these CoV receptors along with immune checkpoint inhibitors in coronavirus positive KIRC patients can be beneficial.

Our study has some limitations as our findings are based on correlation and associations drawn on the analysis of data extracted from several public databases. Further experiments are warranted to confirm the role of CoV receptors in immune modulation of renal carcinoma.

To our best knowledge, this is the first study that analyzed all coronavirus receptors in various cancer types. In brief, our bioinformatics analysis revealed that renal carcinoma patients might be more susceptible to CoV infection. We found evidence that TMPRSS2 may not be the auxiliary protein for SARS-CoV-2 or any coronavirus infection in renal carcinoma. Increased expression of coronavirus receptors suggested that renal carcinoma patients are at increased risk of case related fatalities than healthy subjects. ACE2, DPP4, ANPEP, and ENPEP were associated with a high level of immune infiltration, inflammatory chemokines, cytokines, and markers of an immunosuppressive microenvironment and T cell exhaustion in KIRC tumors. Our study indicates that CoV receptors may play an important role in modulating the immune infiltrate and hence cellular immunity in renal carcinoma and targeting these receptors along with immune-modulating drugs in coronavirus positive patients can be the new treatment modality.

## Methods

### Gene Expression Analysis

We downloaded RNA-Seq gene expression profiling datasets from the Genotype-Tissue Expression study (GTEx Consortium) for human normal tissues (*n* = 8,587). The normalized RNA-Seq data in Transcripts per Million (TPM) was utilized for further analysis. We also downloaded RNA-Seq gene expression profiling datasets from The Cancer Genome Atlas (TCGA) (https://www.cancer.gov/aboutnci/organization/ccg/research/structural-genomics/tcga) (RSEM normalized) for cancer tissues (*n* = 9,736).

### Protein Expression Analysis

The Human Protein Atlas database (https://www.proteinatlas.org/humanproteome) includes expression profiles of RNA and protein corresponding to ~80% of the human protein-coding genes of specific tissues and organs. The protein expression from immunohistochemistry data includes 144 samples, corresponding to 48 different normal human tissue types and 432 tumor samples from 216 different cancer patients. We downloaded the immunohistochemistry (IHC) data analysis of CoV receptors protein in cancer and normal tissues (Björling et al., 2008; Pontén et al., 2009). The score of IHC-based protein expression was determined as the fraction of positive cells defined in different tissues: 0 = 0–1%, 1 = 2–25%, 2 = 26–75%, 3 > 75% and intensity: 0 = negative, 1 = weak, 2 = moderate, and 3 = strong staining. We utilized the combined data of positive fraction and intensity, which is represented as high (3), moderate (2), low (1), or no (0) staining. The IHC data representation for normal tissues was presented as staining of the protein. For carcinoma tissues, we used the percentage positivity based on tumor tissues with high or moderate staining compared to low or no staining detected. Representative images of normal kidney and renal tumor tissues were also acquired from the Human Protein Atlas (https://www.proteinatlas.org/humanproteome).

For analyses of interactions among CoV receptors, the STRING database, which enables analysis for the structural and functional component of proteins (Szklarczyk et al., 2017) was used. The sources for establishing the interactions and enrichment of molecular, functional processes were Text mining, Experiments, Databases, Co-expression, Neighborhood, and Co-occurrence, and 0.4 was set as the cut-off criterion.

### Immune Infiltrate and Subtype Analysis

The Tumor Immune System Interaction database (TISIDB) (http://cis.hku.hk/TISIDB/) platform was used to analyze the correlation of ACE2 with other CoV receptors (DPP4, ANPEP, ENPEP, TMPRSS2) expression in renal carcinoma subtypes (KIRC, *n* = 515; KIRP, *n* = 279; KICH, *n* = 65). The correlation and association of Cov receptors with tumor stage, molecular subtypes, and immuno-subtypes of renal carcinoma were also analyzed using the TISIDB interface.

The Tumor Immune Estimation Resource (TIMER) (https://cistrome.shinyapps.io/timer/) platform comprised of the immune infiltrate data from the TCGA patients (Li et al., 2016, 2017). We investigated the association between CoV receptors expression and the infiltration level of B cell, CD4+ T cell, CD8+ T cell, neutrophil, macrophage, and dendritic cells by gene modules in renal carcinoma subtypes (KIRC, *n* = 553; KIRP, *n* = 290; KICH, *n* = 66). The gene expression level was displayed with log2 RSEM; correlations between CoV receptors expression and genes corresponding to markers of tumor-infiltrating immune cells were explored through correlation modules. The partial Spearman's correlation and statistical significance after purity-correction were shown on the generated scatterplots.

### Immune Signature Enrichment in Renal Carcinoma

To computationally infer the infiltration level of specific immune cell types using RNA-seq data from renal cell carcinoma samples (*n* = 889) and normal renal tissues (*n* = 409) from TCGA, as described previously (Chen et al., 2016), we used a set of 501 genes specifically overexpressed in one of 24 immune cell types (Bindea et al., 2013). We analyzed various immune signatures, including innate immunity, adaptive immunity, pro, and anti-inflammatory cytokines and inflammatory chemokines. The innate immunity marker genes included NK cells, Dendritic cells, Eosinophils, Macrophages, Mast cells, and Neutrophils. The adaptive immunity marker included genes such as B cells, T regulatory cells, T helper cells, and Cytotoxic T cells. We included cytokines like IL1A, IL4, IL6, IL8, IL10, IL11, IL13, TGFB1, and TNF. The chemokines were represented by CCL2, CCL3, CCL4, CCL5, CCL11, and CXCL10.

For pan-cancer correlation analyses, we used the set of 10,224 RNA-seq profiles from 32 different cancer types as featured in our previous study (Chen et al., 2018), using the Bindea immune cell signature scores as computed for this study.

### Statistical Analysis

The correlation among CoV receptors expression and with immune infiltration level was determined by the TIMER interface using Spearman's correlation analysis and statistical significance, and the strength of the correlation was determined using the following guide for the absolute value: 0.00–0.19 “weak,” 0.20–0.59 “moderate,” 0.60–1.0 “strong.” The association between the CoV receptors expression and molecular or immune-subtypes in renal carcinoma was analyzed by the TISIDB interface using the Kruskal–Wallis Test. For comparisons among cancer or normal tissues, Kruskal–Wallis tests with *post hoc* using Dunn's method were performed (GraphPad Prism 7.0 software). For preparing the bar graph, the data was analyzed using GraphPad™ software and presented as mean ± SD. Two-side *p* < 0.05 was considered statistically significant unless otherwise specified.

## Data Availability Statement

All datasets generated for this study are included in the article/Supplementary Material.

## Author Contributions

ST and VD conceived the project and analyzed the data. ST, VD, CC, and AP contributed to the interpretation of the data. All authors wrote and approved the final manuscript.

## Conflict of Interest

The authors declare that the research was conducted in the absence of any commercial or financial relationships that could be construed as a potential conflict of interest.
