# Supplementary material for: Renal Carcinoma Is Associated With Increased Risk of Coronavirus Infections
Source: Front Mol Biosci. 2020 Nov 20;7:579422. doi: 10.3389/fmolb.2020.579422 (PMC7714998; doi:10.3389/fmolb.2020.579422)
Supplement: Supplementary file 1 [file Data_Sheet_1.PDF]

Title: Renal carcinoma is associated with increased risk of coronavirus infections

Authors: Satyendra C Tripathi[1*], Vishwajit Deshmukh[2], Chad J. Creighton[3], Ashlesh Patil[4]

[1]Department of Biochemistry, All India Institute of Medical Sciences, Nagpur, India

[2]Department of Anatomy, All India Institute of Medical Sciences, Nagpur, India

[3]Department of Medicine and Dan L Duncan Comprehensive Cancer Center Baylor College of Medicine, TX, USA

[4]Department of Physiology, All India Institute of Medical Sciences, Nagpur, India

[*]Correspondence

Dr Satyendra C. Tripathi

sctripathi@aiimsnagpur.edu.in

Keywords: Coronavirus, Cancer, Kidney, ACE2, DPP4, TMPRSS2

Supplementary Figure 1: Individual bar graphs for mRNA expression of coronavirus receptors (ACE2, DPP4, ANPEP, ENPEP and TMPRSS2) in different cancer types. RNA-Seq data was extracted from TCGA, RSEM normalized (n=9736). Renal tumors exhibited the highest expression of ACE2 receptor followed by gastrointestinal cancers such as colorectal, pancreatic, and stomach cancer

Supplementary Figure 2: Gene_DE module of TISIDB was used to analyze the differential expression between tumor and adjacent normal tissues. Distributions of coronavirus receptors expression levels across all TCGA tumors are displayed using box plots. Normal tissue data is displayed in gray columns when available. (*: p-value < 0.05; **: p-value <0.01; ***: p-value <0.001).

Supplementary Figure 3: (A) The bar graph for comparison of coronavirus receptor expression across renal cancer types (KIRC, n = 515; KIRP, n = 279; KICH, n = 65) (B) Violin plot for coronavirus receptors expression across various Immune subtypes in KICH and KIRP tumors. (C) Pan-cancer analysis, examining correlations between gene expression-based signatures of immune cell infiltrates (columns) and five different coronavirus related receptor genes (*ACE2*, *ANPEP*, *DPP4*, *ENPEP*, *TMPRSS2*), for each of 32 different cancer types represented in The Cancer Genome Atlas (TCGA). Each matrix entry represents the correlation between the given receptor gene expression and the given immune cell signature, for the given cancer type. Correlations by Pearson's using log-transformed expression values. Purple, high correlation; cyan, low correlation. (D) Heatmaps of differential expression across renal cell carcinoma cases (from TCGA dataset (Chen et al., 2016) for genes encoding ACE2 and DPP4 (top) and genes encoding immunotherapeutic targets in cancer (bottom). Cases are ordered by molecular subtype as defined previously (Chen et al., 2016). Three of these subtypes—CC-e.1, CC-e.2, and CC-e.3—are enriched for KIRC cases; four other subtypes—P-e.1a, P-e.1b, P-e.2, and P.CIMP-e—are enriched for KIRP cases; one subtype, Ch-e, is enriched for KICH cases; and one subtype ("mixed") is not enriched for any of the above. KIRP, Kidney renal papillary cell carcinoma. KIRC, Kidney renal clear cell carcinoma, KICH, Kidney renal chromophobe.

Supplementary Figure 4: Correlation of coronavirus receptors and Tumor immune infiltrate (A, B, C) Correlation scatterplot of (A) TMPRSS2, (B) ANPEP and (C) ENPEP with tumor purity and tumor immune infiltration of B cell, CD8+ T cell, CD4+ T cell, Macrophage, Neutrophil and Dendritic cell in renal carcinoma (KIRC, n = 553; KIRP, n = 290; KICH, n =

66). KICH, Kidney renal chromophobe. KIRP, Kidney renal papillary cell carcinoma. KIRC, Kidney renal clear cell carcinoma.
