# Supplementary figures and images for: Renal Carcinoma Is Associated With Increased Risk of Coronavirus Infections
Source: Front Mol Biosci. 2020 Nov 20;7:579422. doi: 10.3389/fmolb.2020.579422 (PMC7714998; doi:10.3389/fmolb.2020.579422)

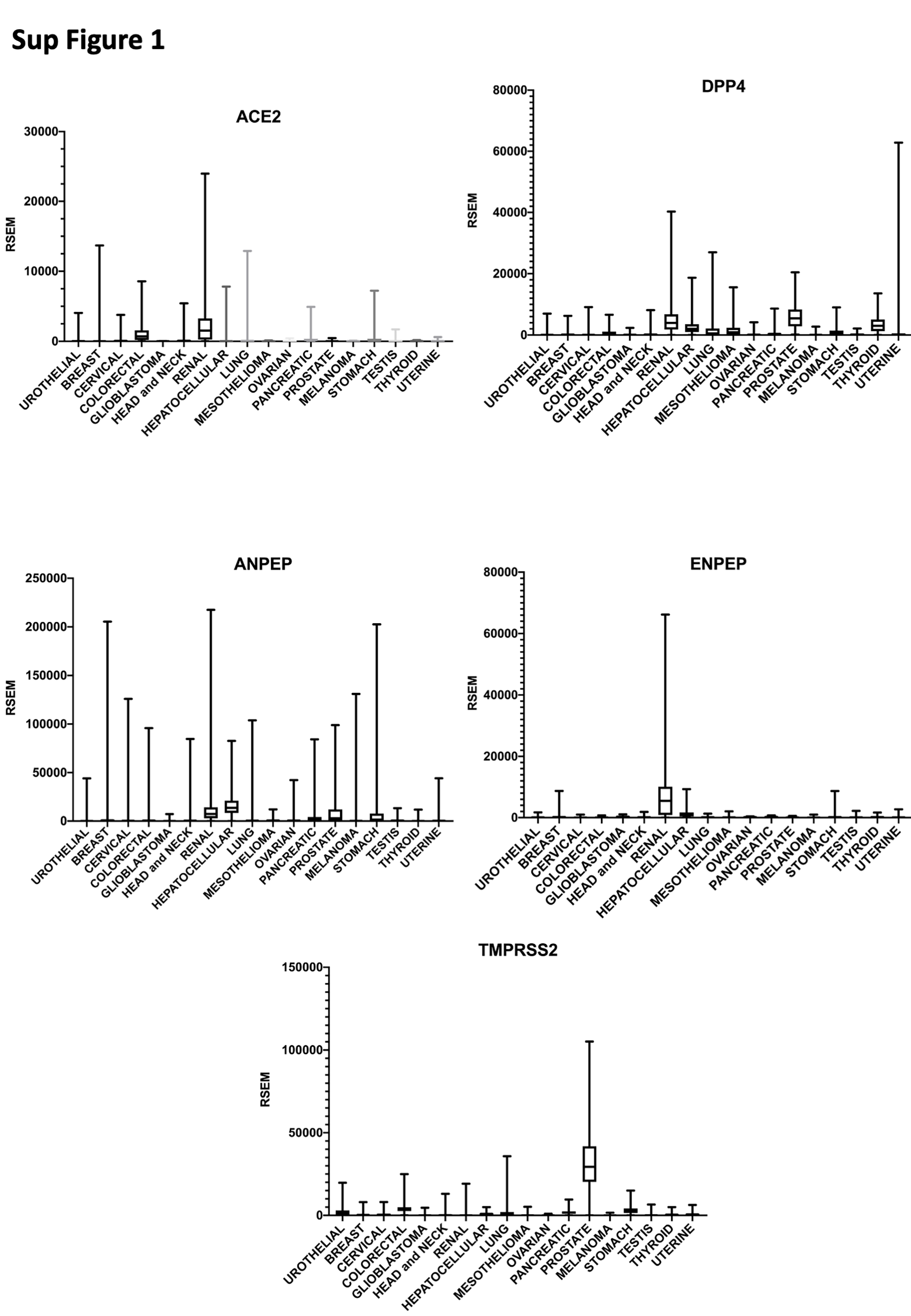

Supplement: Supplementary file 2 [file Image_1.TIFF]

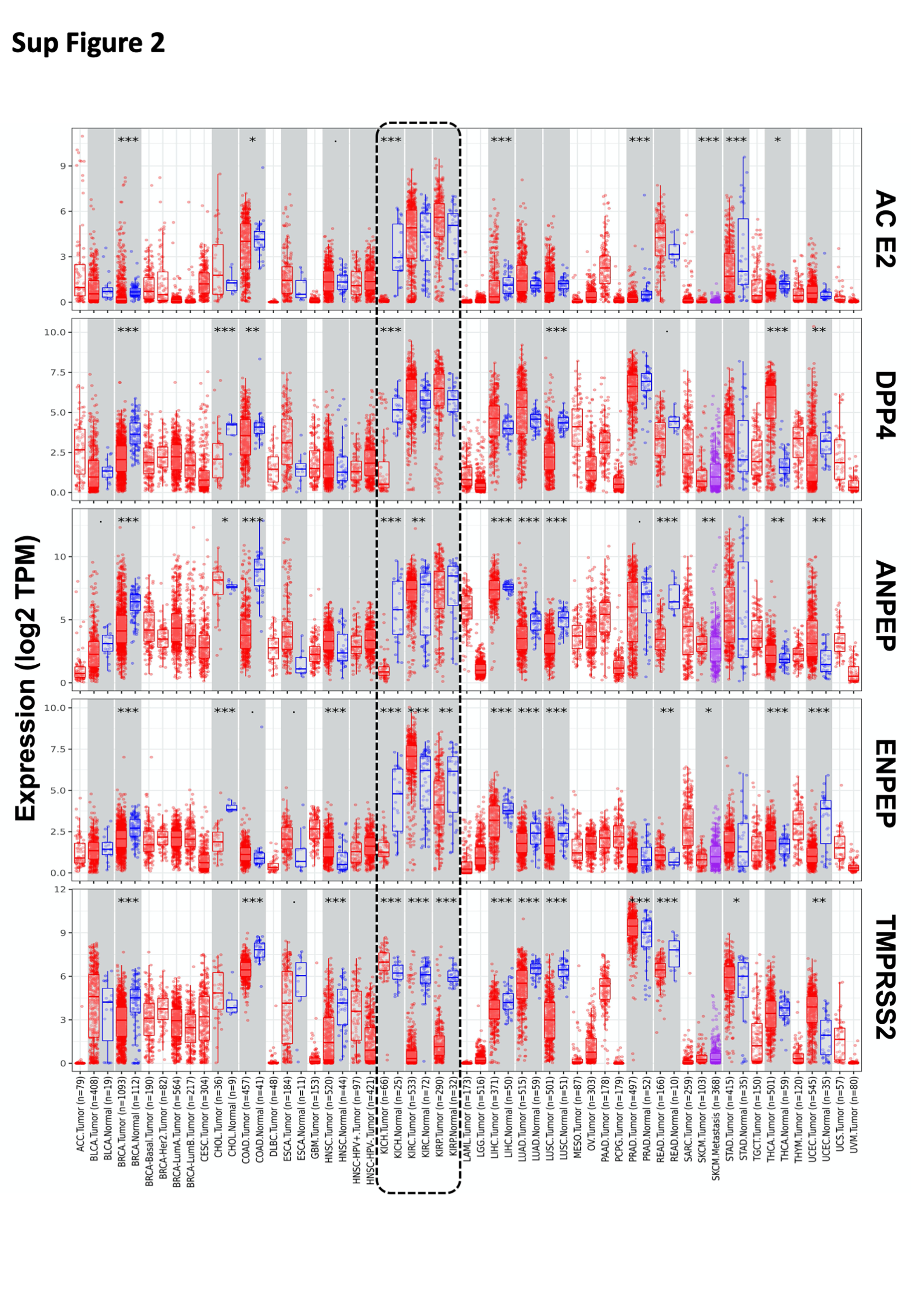

Supplement: Supplementary file 3 [file Image_2.TIFF]

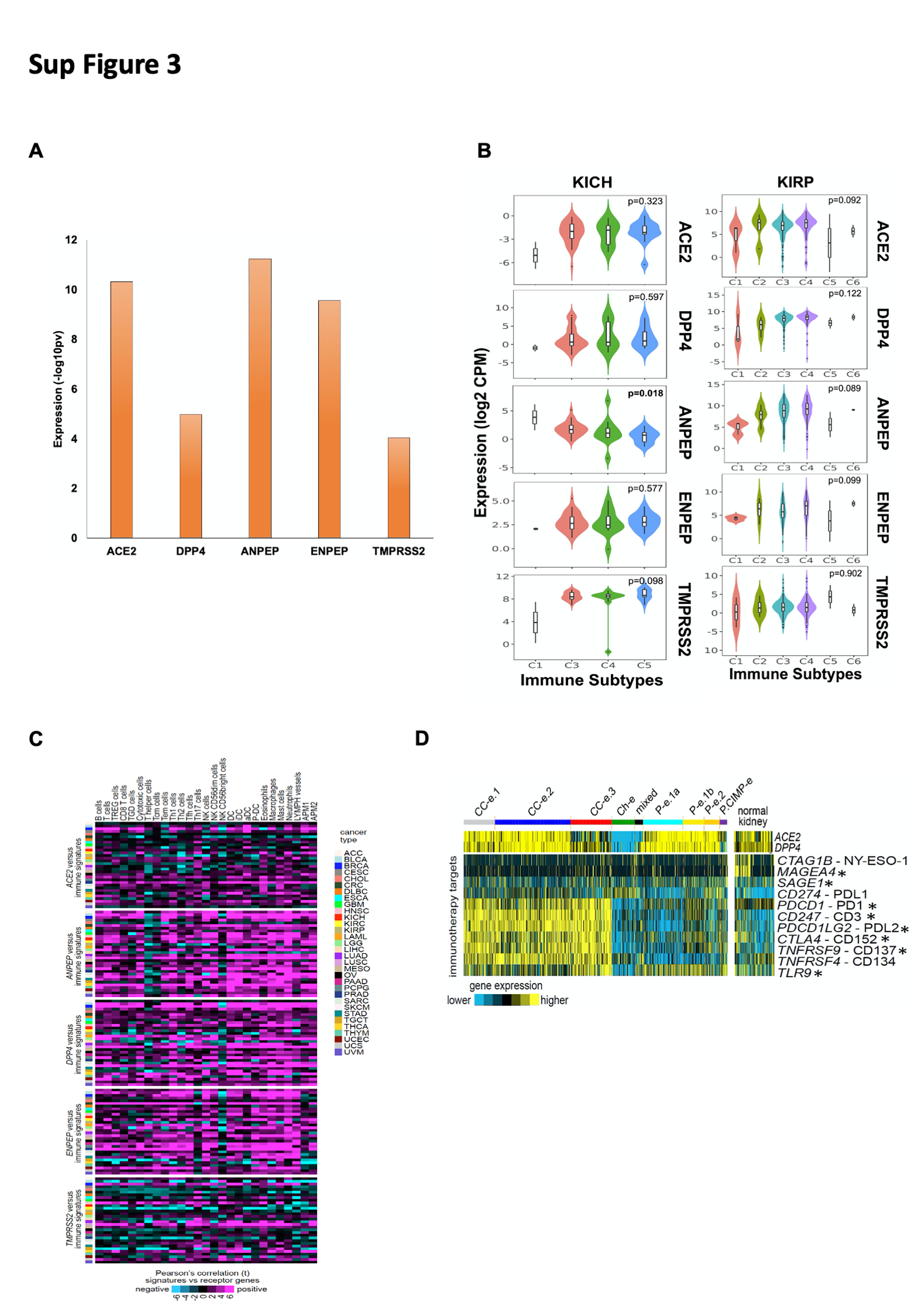

Supplement: Supplementary file 4 [file Image_3.TIFF]

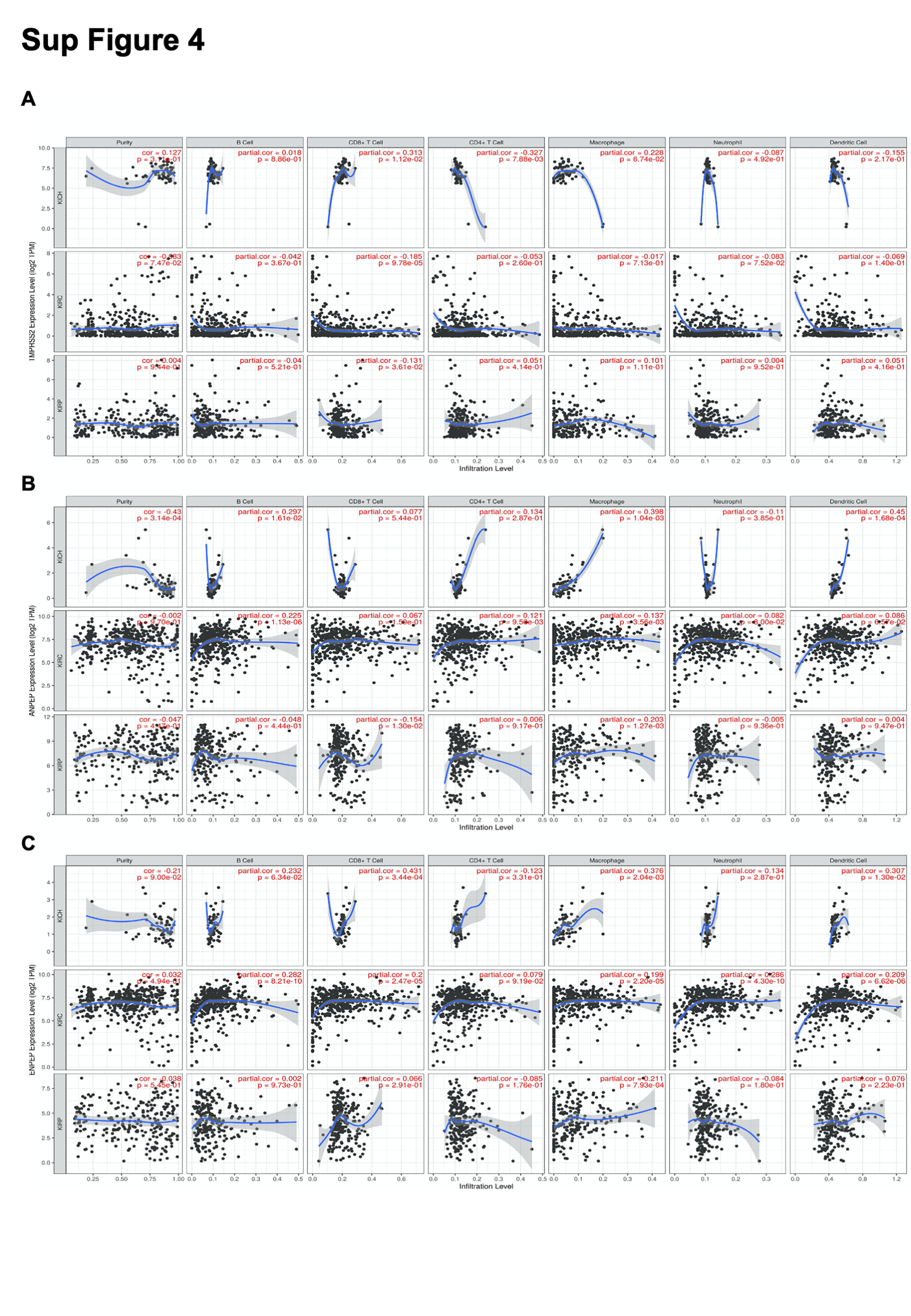

Supplement: Supplementary file 5 [file Image_4.TIFF]
